# Impact of ‘brown rot’ caused by *Gnomoniopsis castanea* on chestnut fruits during the post‐harvest process: critical phases and proposed solutions

**DOI:** 10.1002/jsfa.11397

**Published:** 2021-07-09

**Authors:** Carmen Morales‐Rodriguez, Giorgia Bastianelli, Romina Caccia, Giacomo Bedini, Riccardo Massantini, Roberto Moscetti, Thomas Thomidis, Andrea Vannini

**Affiliations:** ^1^ Department for Innovation in Biological, Agro‐food and Forest systems (DIBAF) University of Tuscia Viterbo Italy; ^2^ Department of Human Nutrition and Diabetics International Hellenic University Thessaloniki Greece

**Keywords:** *Castanea sativa*, internal decay, sterilization, fungal contamination, sensory qualification

## Abstract

**BACKGROUND:**

The brown rot fungus, *Gnomoniopsis castanea*, is the main organism responsible for the outbreak of chestnut postharvest decay that is threatening the sustainability of the chestnut market in Europe. Currently, no specific strategy is available to mitigate the impact and remediate the high losses of fruits in postharvest storage. In the present study, the different phases of chestnut handling in a standard facility plant were analyzed by evaluating the amount of fruit rot and infection by *G. castanea* at each phase.

**RESULTS:**

The warm bath (48 °C) was identified as the critical phase, requiring strict parametrization to effectively inactivate *G. castanea* in fruits. Laboratory tests indicated that maintaining fruits at 50 °C for a maximum of 45 min provided optimal conditions to completely inactivate *G. castanea* inoculum during postharvest handling. However, the warm bath at 50 °C and over was not effective in inactivating the complex of fungal taxa responsible for contamination and development of molds. Higher temperatures and extended treatment times caused significant losses in fruit quality, as indicated by taste panel evaluation. Upscaling of postharvest facilities is discussed and critically evaluated.

**CONCLUSION:**

The warm bath (50 °C for 45 min) is effective in completely inactivating *G. castanea* in fruits but did not reduce the impacts of the complex of molds responsible for external contamination and mycotoxin production. © 2021 The Authors. *Journal of The Science of Food and Agriculture* published by John Wiley & Sons Ltd on behalf of Society of Chemical Industry.

## INTRODUCTION

Before the outbreak of the brown rot fungus *Gnomoniopsis castanea*, G. Tamietti (syn. *Gnomoniopsis smithogilvyi* L.A. Shuttleworth, E.C.Y. Liew 26 & D.I. Guest),^1^, ^2^ fruit spoilage caused by insects, fungi, bacteria, and abiotic factors was considered to be one of the problems of the sweet chestnut industry and market in different production regions worldwide, but generally affecting a low percentage of fruits in storage. As a consequence, various nut‐treatment techniques were developed. The most common treatments are water curing (*curatura* in Italian) and thermo‐hydrotherapy (warm bath), developed in Italy during the 1930s.^3^ The *curatura*, known in the past as ‘novena’, because fruits were usually kept in water for 9 days,^4^ is a classical method used for its proven ability to control insect infestation, killing the larvae of pests (mainly *Curculio* spp. and *Cydia* spp.) and molds without influencing nut quality.^5^, ^6^, ^7^ In contrast, *sterilization* consists of submerging the nuts in steel bins containing a warm water at a temperature recommended to be in the range of 48–52 °C for 35–45 min followed by rapid cooling in tap water (c.a. 15 min).^5^, ^8^, ^9^ This method was proposed to kill insect larvae in a shorter time than the *curatura*, although it could also reduce, but not eliminate, the mycoflora contaminating the fruits.^5^, ^8^, ^9^ More recent studies introduced the use of hot air‐assisted radiofrequency treatments as a physical method to reduce the rate of mold contamination of chestnut fruits in storage, specifically targeting *Penicillium* spp.^10^ Studies were published employing a combination of biological and physical methods,^11^ or the use of a range of chemical treatments^12^ targeting contaminating microorganisms. These methods reduced contamination by a range of fungal taxa associated with the spoilage and rot of the product in postharvest, including *Acrospeira mirabilis*, *Alternaria* spp., *Aspergillus* spp., *Botrytis cinerea*, *Sclerotinia pseudotuberosa*, *Colletotrichum acutatum*., *Fusarium* spp., *Mucor* spp., *Penicillium* spp., *Phoma castanea*, *Phomopsis endogena*, *Phomopsis viterbensis*, *Rhizopus* spp., *Trichoderma* spp., and *Trichothecium roseum*.^5^, ^13^, ^14^, ^15^, ^16^, ^17^, ^18^, ^19^

Postharvest problems in sweet chestnut changed in the first decade of the 21st century, when European and Australian chestnut growers first noticed a severe increase in internal rot in the fruits, locally affecting up to 80% of production in particular seasons.^2^, ^20^, ^21^, ^22^ This new disease was named brown rot for its typical symptomatology and was demonstrated to be caused by *G. castanea*, a fungus commonly found as an endophyte in most chestnut tissues and known to cause a range of different symptoms in the genus *Castanea* and other tree species.^19^ The severe impact of the pathogen in the last decade was associated with a massive presence of inoculum in the environment boosted by climate change,^23^ and in synergy with infestation by the Chinese gall wasp *Dryocosmus kuriphilus* Yasumatsu.^24^, ^25^, ^26^ Chestnut rot results in browning and necrosis of the endosperm and embryo.^1^ The symptoms are mainly expressed postharvest, although rotting fruits can be found in burrs on the tree before harvest.^20^ Notably, fruits that appear healthy have latent infections of the pathogen that, in conditions of optimal temperature and presence of water, rapidly colonize the endosperm in postharvest conditions. Quantifying the impact suggests that incidence may be as high as 93.5% in north‐west Italy,^2^, ^23^ 72% in Australia,^22^ and 21% in Switzerland;^27^ the disease was more recently recorded in North America^28^ and Chile (Vannini, unpublished).

The sudden outbreak of chestnut rot exacerbated the problems of fruit spoilage, putting the sustainability of the chestnut market at serious risk in different regions of Europe: ‘business‐as‐usual’ fruit‐handling methods were not able to remediate the strong impact of the disease postharvest. Thus, efforts are required to analyze the commonly employed postharvest handling chain, determining the critical phases that exacerbate the problem, and those that may best contribute to a reduction in brown rot incidence.

The aims of the present study were (i) to evaluate the impact of *G. castanea* and associated brown‐rot symptoms at each phase of handling under standard conditions, and to highlight critical phases in postharvest handling; and (ii) to determine the optimum parameters for sterilization to assure complete and durable inactivation of *G. castanea* inoculum, without affecting the organoleptic characteristics of the fruit.

## MATERIALS AND METHODS

This work was carried out during the 2018 harvest season in one of the largest chestnut‐producing districts in Central Italy, the Monti Cimini area (province of Viterbo, Italy), hitherto characterized by highly productive managed orchards and coppices. The chestnut fruits analyzed in this study were harvested during the 2018 production season and were provided by the Mastrogregori Ltd processing facility (www.mastrogregori.it/, Vallerano, Italy). Chestnut fruits at this facility are passed through the following phases: (P1) delivery to the plant when a rough estimation of overall damage is carried out by visual assessment of 0.1% per batch with a minimum of 1 kg; a first grading is carried out, and the product is separated from impurities by forced air; (P2) *curatura* when the product is immersed in tap water (10–15 °C) for 72–96 h in large bins (ratio fruits: water = 2:1 v/v); (P3) soaking phase, where the product is rapidly immersed in tap water in a large bin – floating fruits and debris are removed; (P4) the sterilization phase (warm bath) at a temperature of 48 °C^5^ for 35–40 min in continuous flow (ratio fruits: water = 1:1 v/v) followed by a cooling phase in tap water (12–17 min) and drying with forced air. The equipment can handle up to 5000 kg h ^−1^ of chestnuts. The water in the warm bath is changed daily and the tank is cleaned. The water in the cooling tank is changed continuously to maintain a low temperature (15 °C); (P5) grading and manual selection before storage in large refrigeration cells at 0 ± 2 °C and 90% ± 1% RH.

### Impact and presence of *Gnomoniopsis castanea* and total fungal community during chestnut handling phases in a chestnut warehouse

#### Evaluation of the presence of *G. castanea* in chestnut fruits

Three fruit samples (500 ± 30 g; ca. 30 fruits per sample) were collected randomly at the end of phases 1 to 5; half of the sample was analyzed at time 0 (T0) and the other half was stored for 30 days (T30) at 0 °C. Fruits were opened, the presence of ‘brown rot’ recorded, and symptomatic fruits were analyzed to check for the presence of *G. castanea*. Isolation in pure culture was carried out as follows: after shell removal, each kernel was split in half with a sterilized razor blade. Five fragments of each symptomatic fruit were placed onto Petri dishes containing potato dextrose agar (PDA) (Oxoid, Basingstoke, UK, 39 g L^−1^) amended with streptomycin sulfate (0.06 g L^−1^) (PDAs) and incubated at 20 ± 1 °C.^29^ After 7 days of incubation, cultures were scored for the presence of *G. castanea* colonies. Cultures were re‐scored after 30 days to confirm the absence of growth on fragments that were scored negative at day 7. Identification was based on the morphology of the colony and reproductive structures and was ultimately confirmed by molecular barcoding using the protocol reported by Morales‐Rodriguez *et al*.^30^ DNA was extracted from fresh mycelium grown in PDA with the NucleoSpin Plant II mini kit (Mackery Nagel, Düren, Germany) following the manufacturers' instructions. The nuclear Internal Trascribed Spacer (ITS) of rDNA was amplified and sequenced from a subset of each sample using the primers ITS4 and ITS1F.^31^ Amplicons were purified with NucleoSpin Gel and PCR Clean‐up (Mackery Nagel, Düren, Germany). Sequencing reactions were performed by Eurofins Genomics Laboratory (Ebersberg, Germany). Forward and reverse sequences were assembled and edited using BioEdit (Ibis Bioscience, Carlsbad, CA, USA) and compared to accessions in the National Center for Biotechnology Information (NCBI) database. Data were expressed based on the percentage of rotted fruits and the percentage of fruit where *G. castanea* was isolated. The experiment was repeated twice.

#### Evaluation of the total fungal community in water during the sterilization and cooling phase

One‐and‐a‐half liters of water were collected from each the sterilization tank (48 °C) and the cooling tank in phase 4. Serial dilutions were prepared using the protocol described by Hirte;^32^ 500 μL of each dilution was plated on PDA; ten dishes per dilution were prepared and the experiment was repeated twice. Fungal colony‐forming units (CFU) were counted after 72 h of incubation at 20 ± 2 °C. Fungal morphotypes were transferred on fresh PDA, incubated for 7 days at 20 ± 2 °C and identified by sequencing the ITS region, as described in the previous subsection.

### Laboratory trials to evaluate the efficacy of warm bath treatment on the survival of *G. castanea* in chestnut fruits

To investigate the impact of the warm bath treatment on the survival of *G. castanea* inoculum, the experimental design included temperature and time as factors. Thus, three temperatures (45, 50, and 54 °C) plus control (20 °C), and three times (30, 45, and 60 min) were tested, for a total of 12 treatments. A circulating water bath was used (TSCIR35, ThermoFisher Scientific, Waltham, MA, USA) with temperature stability of ±0.1 °C and temperature uniformity of ±0.05 °C. There were two replicates of 1 kg (ca. 60 fruits) per treatment. After water treatment, each sample was cooled in cold water (4 °C) for 1 h and air dried until the weight before treatment was attained. After 15 days at 0 °C in a cold room (90% ± 1% RH and airflow 12 m^3^ h^−1^), the rot class from all fruits treated was assessed. The internal rot class was estimated according to a visual scale where 0 = healthy fruit; 1 = rot up to 50%; 2 = rot >50%. For each treatment, six chestnut fruits were randomly sorted from each rot class (0, 1 and 2) and five fragments of each fruit were placed onto Petri dishes containing PDA before incubation at 20 ± 2 °C. After 7 and 30 days of incubation, plates were scored for the presence of *G. castanea* as described above. Data were expressed as the number of *G. castanea* positive fragments.

Furthermore, 10 mL of water used on each treatment was analyzed for the presence of *G. castanea* by serial dilutions, as described above.

### Sensory evaluation of chestnut fruits after warm bath treatment: laboratory trials

A sensory evaluation was carried out to evaluate changes in the main organoleptic traits of fruit after hot‐water treatment. Four temperature treatments (50, 54, 58, and 60 °C) effective in inactivating *G. castanea* inoculum, three times of exposure (30, 45, 60 min) and two storage times (no storage, 2 weeks of storage at 0 °C in cold room) were combined in a randomized block design to obtain 48 different treatments (1 kg of chestnuts per treatment). The quantitative descriptive analysis (QDA) method^33^ was used by a group of ten trained panelists as an analytical‐descriptive method. The members of the panel were similar in experience, and training background. A sensory evaluation environment was set up with white booths and plastic containers to hold each test sample and water. The containers held five fruits and the tasters were invited to eat as many fruits as they wished from each treatment and then to fill in the evaluation. The samples were evaluated from left to right and the order was randomized in each booth. The sensory evaluation took place in a single session and was recorded manually using report forms especially prepared for the test.^34^

Four sensory attributes considered important for consumer acceptance and potentially affected by warm bath treatment^35^ were chosen for evaluation: crispness, bitterness, shell color, and pulp color. Sweetness was also considered as the indication of a higher risk of perishability due to the release of free sugar as a substrate for contamination by saprotrophic micro‐organisms. Qualitative variables of the descriptors were transformed into quantitative variables using a numerical four‐degree scale (Table 1). The evaluation was carried out using untreated chestnuts as controls, for which all descriptors were scored 1.

**Table 1:** Qualitative descriptors used in for sensory analysis

Descriptors	1	2	3	4
Crispness	normal	slightly altered	altered	absent
Bitterness	absent	tolerable	bitter	very bitter
Sweetness	normal	Slightly altered	sweet	very sweet
Shell color	normal	slightly altered	altered	very altered
Pulp color	normal	slightly altered	altered	very altered

### Statistical analysis

Data normality and equal variances were tested using the Kolmogorov–Smirnov and Bartlett tests, respectively. Percentages of brown rot and *G. castanea* isolation in the chestnut‐handling phases fulfilled these assumptions and were further analyzed by a one‐way ANOVA, followed by Tukey's honest significant difference (HSD) post‐hoc tests at the 95% family‐wise confidence level (P < 0.05). To compare between two sets as the percentages of brown rot and *G. castanea* isolation at T0 and T30, and CFUs from water collected from the sterilization tank and the cooling tank in phase 4, an unpaired Student's *t*‐test was performed. All analyses were carried in Prism version 8.00 (GraphPad Software, San Diego, California, USA).

Data from laboratory trials to evaluate the efficacy of hot‐water treatments were not normally distributed; therefore, a general linear model (GLM) was employed^36^ using Statgraphics Centurion XVI, v16.1.18 (Statgraphics.Net, Madrid, Spain). A Welch ANOVA and Games‐Howell's multiple comparison tests were applied to analyze CFUs from water samples in laboratory trials because of significant differences in Bartlett's tests for equal variances.^37^ Analyses were carried out in Prism version 8.00.

Past version 4.0 (Paleontological Statistics Software, Oslo, Norway) was used to obtain a radar chart of the panel test data.

## RESULTS

### Impact and presence of *Gnomoniopsis castanea* and total fungal community during chestnut handling phases in a chestnut warehouse

#### Evaluation of the presence and impact of *G. castanea* in chestnut fruits from standard treatment

**Figure 1:**
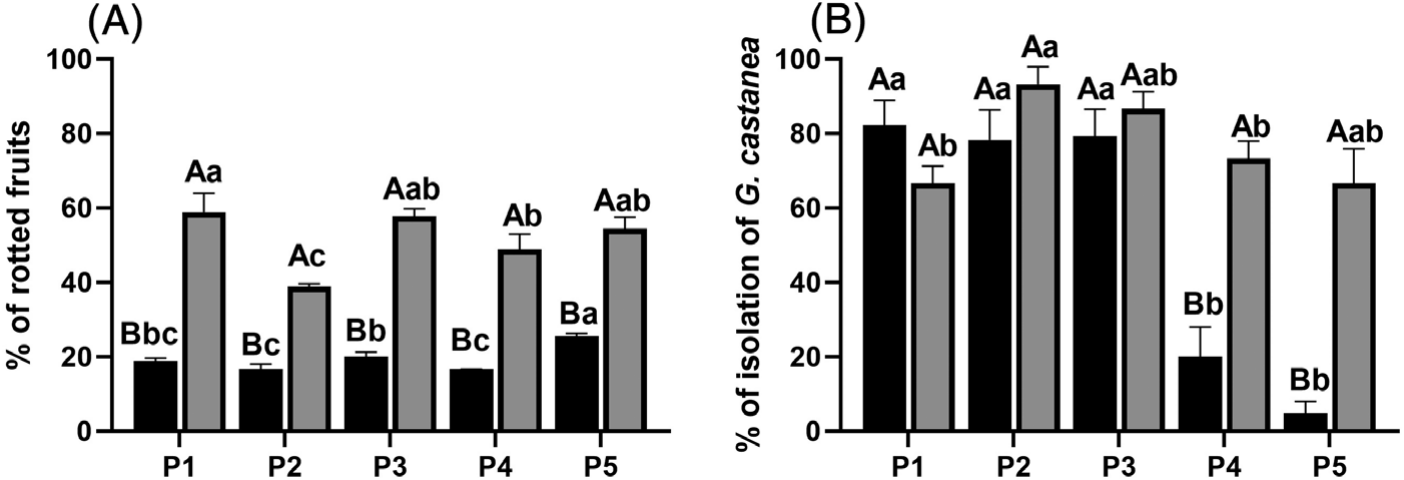
Percentage of brown rot (± SEM) (A) and percentage of isolation of *G. castanea* from rotten fruits (± SEM) of *Castanea sativa* (B) in the five defined phases of postharvest, at time 0 (T0; black bars) and after 30 days of storage at 0 °C (T30, grey bars). The same lower case letters indicate no significant differences among phases (Tukey's HSD post‐hoc test). The same upper case letters indicate no significant differences at each phase between T0 and T30 (unpaired *t*‐test; *P* < 0.001).

The one‐way ANOVA showed significant differences (*P* < 0.001) in the percentage of rotted fruits among phases at both storage times (T0; F = 16.87; T30; F = 7.14). The percentage of fruits with rot increased during the phases of the postharvest handling: treatments carried out in phases 2 and 4 (*curatura* and sterilization, respectively) presented the lower percentage of rot (16%) but this increased to 25% at phase 5 (manual sorting). Tukey's HSD post‐hoc test results are shown in Fig. 1(A). After 30 days of storage, the percentages of brown rot doubled in all phases. In the case of the last phase (manual sorting) corresponding to a commercially ready product, the percentage increased from 25% to 54%. An unpaired *t*‐test between T0 and T30 showed significant differences at each phase, with the percentage of brown rot always higher after 30 days (Fig. 1(A)). In the same way, for the isolation of *G. castanea*, a one‐way ANOVA showed a significant reduction (*P* < 0.001) in the percentage of isolation during the postharvest handling phases analyzed at both times (T0; F = 35.45; T30; F = 5.16). The percentage of isolation was between 82% and 78% for the first three phases of the industrial chain (respectively, P1, P2 and P3) subsequently decreased to 20% for P4 (sterilization) and 5% for P5 (manual sorting) (Fig.1(B)). The effect of the treatments carried out in the handling chain were not permanent and at T30 the percentage of *G. castanea* isolation increased. An unpaired *t*‐test between T0 and T30 at each phase showed a significant (*P* < 0.001) increase in isolation of *G. castanea* after storage at P4 (F = 40) and P5 (F = 47.79).

#### Evaluation of the total fungal community in water during phase 4 (sterilization and cooling)

**Figure 2:**
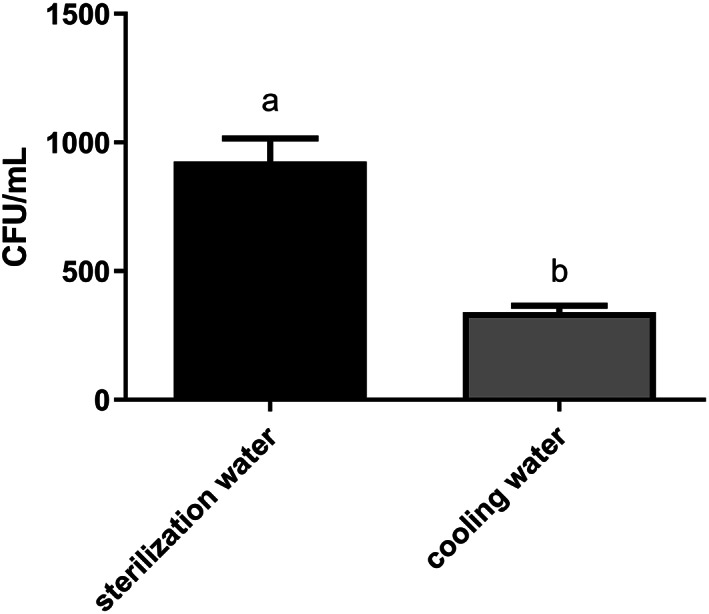
Fungal colony‐forming units (CFU mL^−1^ ± SEM) in 50 °C sterilization water (black bar) and cooling water (gray bar). Values followed by different letters differ significantly (unpaired *t*‐test, *P* < 0.0001).

Sterilization water contained a significantly higher number of fungal CFUs than cooling water (unpaired *t*‐test, *P* < 0.0001), with values of 926 and 340 CFU mL^−1^, respectively (Fig. 2).

The same fungal taxa (8) were identified from both sterilization and cooling water, including *Penicillium echinulatum*, *P. glandicola*, *P. glabrum*, *Trichoderma orientale*, then *Cladosporium cladosporoides*, *Geotrichum candidum*, *Talaromyces amestolkiae*, and several Mucoraceae. *Gnomoniopsis castanea* was never detected.

### Laboratory trials to evaluate the efficacy of hot‐water treatment on the survival of *G. castanea* in chestnut fruits

Treatments at 50 and 54 °C resulted in the total and permanent inactivation of *G. castanea*: the pathogen was not isolated from any fruits that were analyzed, regardless of the storage time and rot class. Thus, these two treatments were not considered in the following statistical analysis. The GLM, with *G. castanea* isolation as dependent variable and temperature, time (30, 45, 60 min), and rot class (0, 1, 2) as categorical factors, was significant (F = 9.56; *P* < 0.0001) (Table 2).

**Table 2:** General linear model (GLM) for *G. castanea* isolation with water temperature, time of treatment, and rot class, as categorical factors

*Source*	*Sum of squares*	*df*	*Mean sum of squares*	*F‐test*	*P‐value*
Temperature	100.463	1	100.463	48.05	**<0.0001**
Rot class	75.6594	2	37.8297	18.09	**<0.0001**
Time	0.366072	2	0.183036	0.09	0.9162
Temperature * rot class	33.6612	2	16.8306	8.05	**<0.001**
Temperature *time	1.47767	2	0.738 837	0.35	0.7030
Rot class * time	6.36837	4	1,59209	0.76	0.5521
Temperature * rot class * time	4.49737	4	1.12434	0.54	0.7082
Residual	275.982	132	2.09077		
Total	54.824	149			

**Figure 3:**
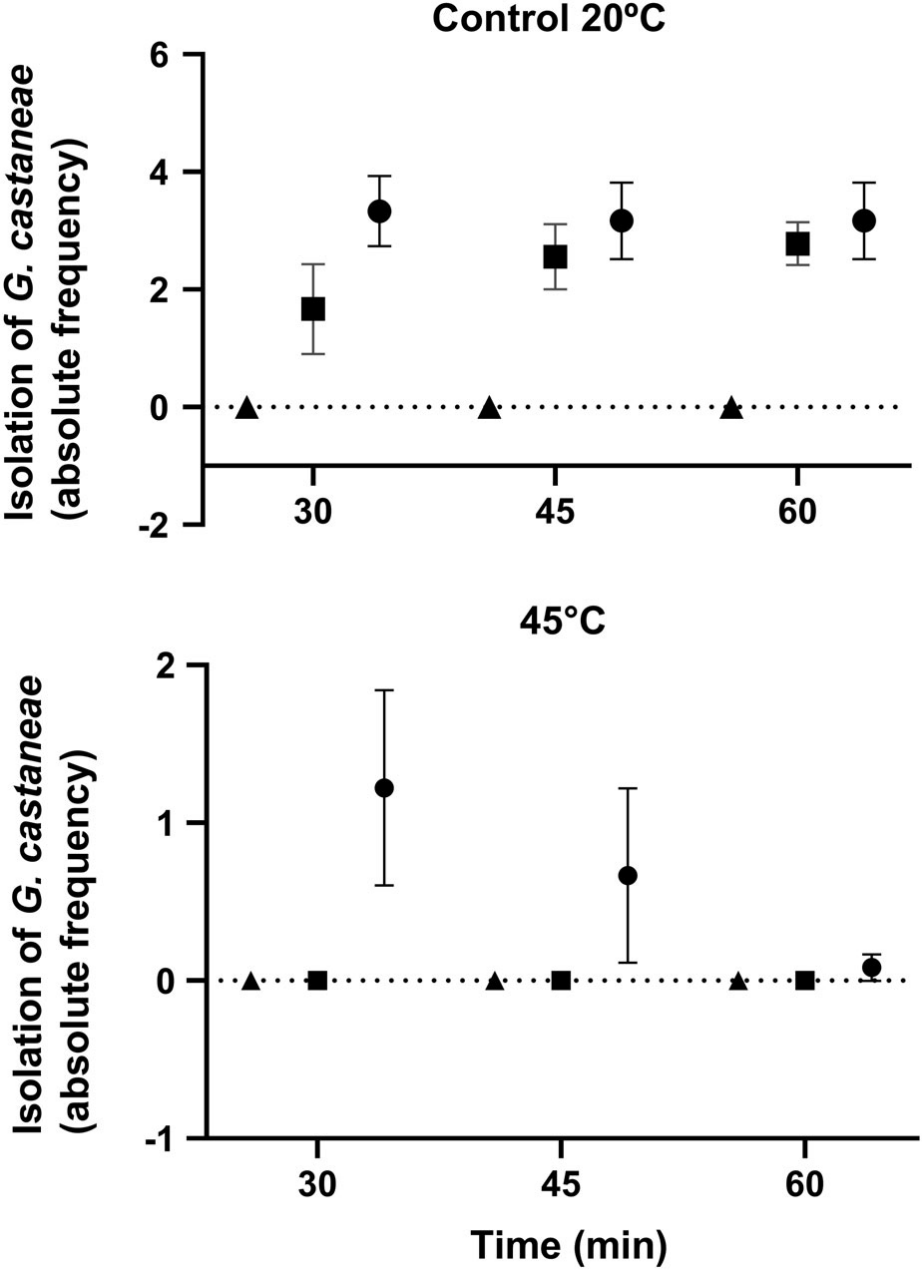
Absolute frequency of *G. castanea* isolation from fruits in rot classes 0 (no‐symptomatic, triangle), 1 (up to 50% of rotted pulp, square), 2 (rotten pulp over 50%, circle) at control temperature (20 °C) (A) and 45 °C (B). For each time of treatment, points with the same letter were not significantly different (*P* > 0.05) according to Tukey's test. Error bars indicate SEM.

However, because of the significant temperature × rot class interaction (*P* = 0.0005), GLMs were performed for each temperature (20 and 45 °C) with time and rot class as categorical factors. At 20 °C, the GLM was significant (F = 4.30; *P* < 0.001). Rot class was a significant factor (F = 16.24; *P* < 0.001), whereas time and the interaction rot class × time interaction were not (F = 0.03 and F = 0.52 respectively; *P* > 0.05). Tukey's HSD post‐hoc test showed significant differences between rot class 0 (healthy fruits) and rot classes 1 and 2 (Fig. 3(A)). At 45 °C, the GLM was also significant (F = 2.20; *P* < 0.05). The rot class was significant (F = 2.21; *P* < 0.005), but time and the interaction rot class × time were not (F = 1.03 and F = 1.21 respectively; *P* > 0.05). Tukey's HSD post‐hoc test showed significant differences between the rot class 0 and classes1 and 2 (Fig. 3(B)).

**Figure 4:**
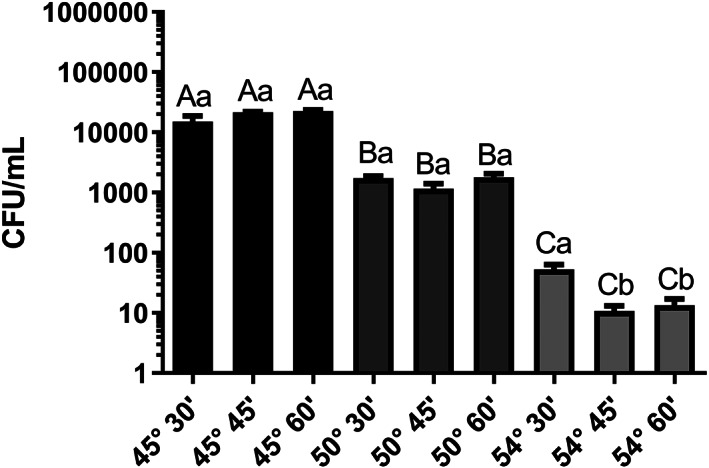
Fungal CFUs from warm‐bath treatments (45°, 50° and 54 °C) of chestnut fruits. Different upper case letters indicate significant differences between temperatures at each time (Games–Howell multiple comparison test). Different lowercase letters indicate significant differences between time at each temperature (Games–Howell multiple comparison test). Error bars indicate SEM.

A presence of fungal CFUs was detected in all warm bath treatments although in decreasing numbers from 45 °C to 54 °C (Fig. 4). For each time (30, 45, 60 min) significant differences were found between temperatures: 30 min (W = 13.89; *P* = 0.001); 45 min (W = 13.89; *P* = 0.0018), and 60 min (W = 72.04; *P* < 0.0001).

A total of seven morphotypes were identified, three of which (*Penicillium echinulatum*, *Galactomyces geotrichum*, and members of the Mucoraceae) represented 79.3, 13.5, and 6.9% of the total CFU. *Gnomoniopsis castanea* was not detected from sterilization water in any temperature, time, or rot class.

### Sensory evaluation of chestnut

**Figure 5:**
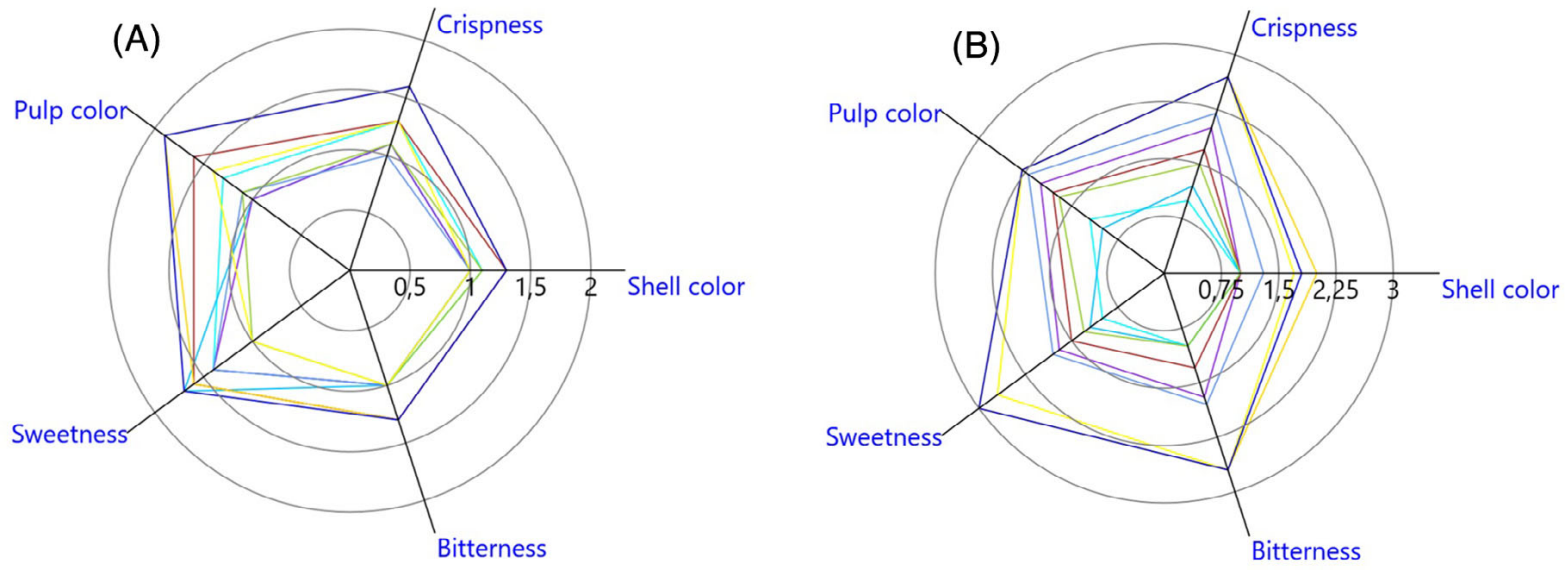
Radar chart of the sensory evaluation of chestnut fruits for each descriptor at T0 (no storage; A) and T15 (after 15 days of storage at 0 °C; B).

The values for each descriptor are shown in a radar chart at T0 (a) and T15 (b) (Fig. 5). At T0, differences compared with the control (fruits in water at 20 °C, T0, descriptors reference value = 1) were evident but within 40% deviation, except for the treatments at 58 °C for 45 and 60 min, where differences were over 50%. At T15, the fruits treated at 50 °C for 30 and 45 min deviated from the controls (fruits in water at 20 °C, T15, descriptors reference value = 1) by 4% and 6% respectively, whereas deviations were much higher for the other treatments, ranging from 30% to 154%. The results of the sensory evaluation of chestnut fruits are reported as a single descriptor and the total score for each temperature and time of treatment are available in the supplementary information Table S1.

## DISCUSSION

In the present study, an assessment of the impact of *G. castanea* on the quality of the fruits in postharvest during each of the phases of handling was carried out in a facility in central Italy. This analysis provided very useful, detailed information on the importance of specific phases in mitigating or exacerbating the impact of the disease. It is important to highlight that halting the progression of the disease relies on irreversible inactivation of the pathogen inoculum. Assuming efficient and continuous sanitation of the handling plant, active inoculum is mostly introduced with sweet chestnuts in the delivery phase (P1). On delivery, there is a rather high proportion of rotted fruits, as determined by destructive mycological analyses of fruit samples; nevertheless, some rotten fruits enter the plant and pass through all the postharvest handling phases, thereby introducing active inoculum of *G. castanea*. In the second phase (*curatura*), the sanitation mechanism is attributed to the partial lactic and alcoholic fermentation taking place during the treatment, which results in a reduction in pH, and an increase in alcohol and acetaldehyde content, coupled with a probable diffusion of phenol compounds from the pericarp to the seed.^4^ The present study suggested that the cold bath did not inactivate the *G. castanea* inoculum. Moreover, there was no fungistatic effect either, as the percentage of isolation of *G. castanea* was not significantly different from that of the previous phase. The soaking phase (P3) also did not affect the proportions of rotten fruits or *G. castanea* isolation frequency. The soaking phase enables the detection and removal of debris and floating chestnuts, commonly those damaged by insects. Chestnuts affected by brown rot, even at an advanced stage of rot, do not float on water. In contrast, P4 drastically reduced the percentage isolation of *G. castanea*, although it did not result in total inactivation of inoculum in the running conditions at the handling plant (48 °C; 40 min). The effect was more fungistatic than fungicidal because after 30 days storage isolation frequency returned to the values obtained immediately after phase 1. As also reported by Jermini *et al*.,^5^ the warm bath (45–48 °C) did not suppress the complexity of mold species present in the chestnuts, some of which also produce mycotoxins; the molds were easily recovered from both the warm bath and the cold bath of the cooling phase. These taxa were the same species or co‐generic with those responsible for causing molds in chestnuts, including *Penicillium* spp.^38^ Several previous publications reported work aiming to develop sustainable chemical and biological treatments of chestnuts for postharvest treatments to reduce fruit spoilage.^11^, ^12^, ^38^, ^39^ The most effective techniques must be considered in the near future to control the complex communities of organisms other than *G. castanea* responsible for fruit spoilage.

The fungistatic effect resulting from P4 continued into the grading and manual selection phase. This phase did not significantly affect both the percentage of rotted fruits or the frequency of isolation of *G. castanea*. The whole process in the facility plant did not reduce the impact of *G. castanea*, as demonstrated by the severe and significant increase in the percentage of rotted fruits after 30 days of storage at 0 °C. The *sterilization* phase is the crucial stage for further work to optimize the process and thereby reduce the impact of *G. castanea* in chestnuts postharvest, thus increasing storability, safety, and shelf‐life of the fruit. Laboratory tests suggested that a warm bath at 50 °C for 30 or 45 min was sufficient to inactivate *G. castanea* permanently, in chestnuts regardless of the level of colonization and rot stage. Longer periods and higher temperatures were also effective, but negatively impacted the sensory characteristics of the fruits. Lower temperatures, 45 °C, effectively inactivated *G. castanea* inoculum in fruits with rot symptoms, but when affecting less than 50% of the pulp. With greater amounts of rot, this temperature did not permanently inactivate the pathogen, regardless of the length of the treatment (30, 45, or 60 min). This point is of particular relevance in the general context of the control of brown rot. As reported above, the *sterilization* tanks in a modern chestnut postharvest facility process several tons of chestnuts per hour in a continuous flow. The mass of fruits enters the *sterilization* phase from the cold bath and the floating phase, at a temperature of approximately 15 °C. To guarantee the efficacy of the warm bath treatment, the mass of fruits must first be taken to the temperature of 50 °C and subsequently maintained at this temperature throughout the fruit mass for at least 30 min. Such conditions are relatively easy to be set and monitor in laboratory conditions but upscaling to the production facility makes monitoring and setting of strict parameters more difficult, depending on the time required to bring the whole mass to the correct temperature and to maintain that temperature for the proper time. It is important to maintain the treatment for sufficient time to totally inactivate the *G. castanea* inoculum in fruits, as a fungistatic effect would not prevent the outbreak of the disease in the storage phase. It must also be considered that a processing system working in continuous flow must synchronize the different phases to guarantee the economic sustainability of the process itself. It might be argued that an increase in the temperature would better guarantee the inactivation of the pathogen and reduce the time of the treatment. However, in the present study, it was evident that treatments at temperatures higher than 50 °C for longer than 45 min irreversibly compromised the sensory quality of the fruits. The sensitivity of fruits to heat treatments strictly depends on the varieties of chestnut in addition to the parametrization of the treatment and the storage conditions.^40^ In general, treatments <42 °C preserve cell integrity even if the enzymatic activity related to ripening and maturation of the fruit are altered. Temperatures >45 °C are considered near the threshold of cell damage with a limited recovery ability.^40^ There are no studies available on the specific temperature and conditions causing cell damage in chestnut fruits but it is likely that at a temperature higher than 50°, a change in pulp color, loss of tissue consistency, and increasing bitterness were indicative of the occurrence of cell damage and the consequent release and oxidation of cell components such as phenol compounds. The application of appropriate protocols to control of ‘brown rot’ in chestnut orchards would probably enable more efficient postharvest treatment. Control methods able to reduce the endophytic inoculum of *G. castanea in planta* and the severity of the symptoms on fruits before delivery to the handling facility would reduce the risk that a high fraction of severely rooted fruits (over 50% of the pulp) enters handling. Activities are under way in different European chestnut areas to develop protocols for the control of ‘brown rot’ in orchards and to find alternatives to compounds currently recommended for use on chestnut (https://www.sancast.it/news-pagina.asp?id=892).

The effectiveness of product sterilization may be improved by selectively removing unsound chestnut fruits from the stock entering the handling plant using non‐destructive methods based on near infrared (NIR) spectroscopy^41^, ^42^ or X‐ray computed tomography.^43^ In fact, recent studies on the feasibility of Fourier transform near infrared (FT‐NIR) spectroscopy or visible‐near infrared hyperspectral imaging in detecting ‘brown rot’ of chestnut fruits demonstrated the feasibility of the approach^44^ and the need for an upscale from prototype to commercial equipment. A molecular tool for the sensitive detection and quantification of *G. castaneae* in symptomatic and asymptomatic fruits has been developed to quantify the *G. castaneae* inoculum.^45^ Furthermore, a recent study by Vettraino *et al*.^46^ provided promising evidence on the use of ozone in storage conditions, specifically as a fungistatic option to avoid increasing chestnut rot in stored stocks. It is clear that an integrated protocol must be developed including the most promising methodologies and technologies to be applied in postharvest handling.

## CONCLUSIONS

In conclusion, the present study demonstrated that application of strict conditions at the *sterilization* phase of chestnut handling can definitively inactivate *G. castanea* in non‐symptomatic and rotting fruits. Sterilization at 50 °C and over was not effective in inactivating the complex of fungal taxa responsible for contamination and development of molds, many of which produce mycotoxins. The efficacy of the warm bath strictly depends on the quality of the fruit stocks entering the handling plant and the conditions to which they are exposed before the P4 phase. An efficient strategy for the control of ‘brown rot’ must include mitigation protocols applied in the open field, followed by effective physical treatments in the handling plant.

The positive results obtained in this work, albeit preliminary, were practical to a targeted application of low‐impact containment methods for *G. castanea* and represent a promising step forward for harvest technologies employed in chestnut handling facilities. However, further studies are needed, both at laboratory and at industrial plant level, to develop a better understanding of the critical points of fungal contamination in the chestnut treatment chain.

## Supporting information


**Table S1.** Individual score for each descriptor and total score of the sensorial evaluation of chestnut fruits following hot‐water treatments for different times. The last column expresses the deviation in percentage from the control whose score for each descriptor was fixed at 1.
