# Supplementary material for: Impact of ‘brown rot’ caused by Gnomoniopsis castanea on chestnut fruits during the post‐harvest process: critical phases and proposed solutions
Source: J Sci Food Agric. 2021 Jul 9;102(2):680–7. doi: 10.1002/jsfa.11397 (PMC9290021; doi:10.1002/jsfa.11397)
Supplement: Supplementary file 1 — Table S1. Individual score for each descriptor and total score of the sensorial evaluation of chestnut fruits following hot‐water treatments for different times. The last column expresses the deviation in percentage from the control whose score for each descriptor was fixed at 1. [file JSFA-102-680-s001.docx]

Table S1. Individual score for each descriptor and total score of the sensorial evaluation of chestnut fruits following hot-water treatments for different times. The last column expresses the deviation in percentage from the control whose score for each descriptor was fixed at 1.

| Storage (days) | Temp (°C) | Time (min) | Individual score | | | | | Total score | Deviation  from control |
| --- | --- | --- | --- | --- | --- | --- | --- | --- | --- |
|  |  |  | Shell color | Crispness | Pulp color | Sweetness | Bitterness |  | (%) |
| none | 50 | 30 | 1,1 | 1,3 | 1,3 | 1,4 | 1,0 | 6.1 | 22 |
|  |  | 45 | 1,0 | 1,1 | 1,0 | 1,7 | 1,0 | 5,9 | 18 |
|  |  | 60 | 1,0 | 1,1 | 1,0 | 1,4 | 1,0 | 5,6 | 12 |
|  | 54 | 30 | 1,3 | 1,3 | 1,6 | 1,6 | 1,3 | 7,0 | 40 |
|  |  | 45 | 1,0 | 1,0 | 1,1 | 1,4 | 1,0 | 5,6 | 12 |
|  |  | 60 | 1,1 | 1,1 | 1,1 | 1,0 | 1,0 | 5,4 | 8 |
|  | 58 | 30 | 1,0 | 1,3 | 1,4 | 1,0 | 1,0 | 5,7 | 14 |
|  |  | 45 | 1,3 | 1,6 | 1,9 | 1,6 | 1,3 | 7,6 | 52 |
|  |  | 60 | 1,3 | 1,6 | 1,9 | 1,7 | 1,3 | 7,7 | 54 |
| 15 | 50 | 30 | 1,0 | 1,0 | 1,2 | 1,0 | 1,0 | 5,2 | 4 |
|  |  | 45 | 1,0 | 1,2 | 1,0 | 1,2 | 1,0 | 5,3 | 6 |
|  |  | 60 | 1,0 | 2,0 | 2,0 | 1,7 | 1,7 | 8,3 | 66 |
|  | 54 | 30 | 1,0 | 1,7 | 1,8 | 1,5 | 1,3 | 7,3 | 46 |
|  |  | 45 | 1,3 | 2,2 | 2,2 | 1,8 | 1,8 | 9,3 | 86 |
|  |  | 60 | 1,0 | 1,5 | 1,7 | 1,3 | 1,0 | 6,5 | 30 |
|  | 58 | 30 | 1,7 | 2,7 | 2,3 | 2,7 | 2,7 | 12,0 | 140 |
|  |  | 45 | 2,0 | 2,7 | 2,3 | 3,0 | 2,7 | 12,7 | 154 |
|  |  | 60 | 1,8 | 2,7 | 2,3 | 3,0 | 2,7 | 12,5 | 150 |
